# Strategy for Treating Motor Neuron Diseases Using a Fusion Protein of Botulinum Toxin Binding Domain and Streptavidin for Viral Vector Access: Work in Progress

**DOI:** 10.3390/toxins2122872

**Published:** 2010-12-20

**Authors:** Daniel B. Drachman, Robert N. Adams, Uma Balasubramanian, Yang Lu

**Affiliations:** 1 Department of Neurology, Johns Hopkins School of Medicine, 600 North Wolfe Ave, Baltimore, MD 21287, USA; Email: rna@jhmi.edu (R.N.A.); uma1@jhmi.edu (U.B.); 2 Sirnaomics, Inc., 401 Professional Dr. Suite 130, Gaithersburg, MD 20879, USA; Email: alanlu@sirnaomics.com

**Keywords:** botulinum toxin, binding domain, motor neuron diseases, ALS, SMA, motor neurons, therapeutic targeting, gene transfer, viral vectors

## Abstract

Although advances in understanding of the pathogenesis of amyotrophic lateral sclerosis (ALS) and spinal muscular atrophy (SMA) have suggested attractive treatment strategies, delivery of agents to motor neurons embedded within the spinal cord is problematic. We have designed a strategy based on the specificity of botulinum toxin, to direct entry of viral vectors carrying candidate therapeutic genes into motor neurons. We have engineered and expressed fusion proteins consisting of the binding domain of botulinum toxin type A fused to streptavidin (SAv). This fusion protein will direct biotinylated viral vectors carrying therapeutic genes into motor nerve terminals where they can enter the acidified endosomal compartments, be released and undergo retrograde transport, to deliver the genes to motor neurons. Both ends of the fusion proteins are shown to be functionally intact. The binding domain end binds to mammalian nerve terminals at neuromuscular junctions, ganglioside GT1b (a target of botulinum toxin), and a variety of neuronal cells including primary chick embryo motor neurons, N2A neuroblastoma cells, NG108-15 cells, but not to NG CR72 cells, which lack complex gangliosides. The streptavidin end binds to biotin, and to a biotinylated Alexa 488 fluorescent tag. Further studies are in progress to evaluate the delivery of genes to motor neurons *in vivo*, by the use of biotinylated viral vectors.

## 1. Introduction

Amyotrophic Lateral Sclerosis (ALS) is a universally fatal and remarkably common disease, with a lifetime risk of developing the disease estimated at between 1 in 400 [1] and 1 in 2,000 [2]. ALS is due to selective loss of motor neurons, resulting in progressive muscular weakness, denervation atrophy and spasticity. The typical age of onset is 50 to 60 years, and death generally occurs within 5 years, usually due to respiratory failure. Spinal Muscular Atrophy (SMA) is a recessively inherited disorder that also results in loss of motor neurons. In its most severe form, it occurs in infancy, and is the leading genetic cause of infant death, with an estimated incidence of 10–16 in 100,000 live births. SMA also causes milder forms of the disease with weakness developing in childhood or later. The carrier frequency of the recessive genetic abnormality in SMA is 1 in 40 to 1 in 50 [3,4].

Although advances in knowledge of the pathogenesis of these motor neuron diseases have suggested several attractive candidate treatment strategies, a key problem in therapy of ALS and SMA is the enormous difficulty of delivering agents to the motor neurons, which are embedded within the spinal cord and are relatively inaccessible. To attempt to solve this problem we have been developing a strategy based on the absolute neurospecificity of botulinum toxin, to direct the entry of viral vectors carrying candidate therapeutic genes into motor neurons. We have engineered and expressed fusion proteins consisting of the binding domain of botulinum toxin type A fused to streptavidin (SAv). The goal is to utilize the botulinum-SAv fusion protein as a “Trojan horse” to direct biotinylated viral vectors carrying candidate therapeutic genes into motor nerve terminals. The binding domain of the Botulinum toxin heavy chain (*i.e.*, the C-terminal end) specifically directs binding to, and entry into, motor nerve terminals. The SAv part of the fusion protein binds tightly to biotin, and in this strategy is designed to bind biotinylated viral vectors. The endocytosed fusion protein-viral vector complexes then enter the acidified endosomal compartments, from which the viruses will be released and undergo retrograde transport, where they are expected to deliver the potentially therapeutic genes to the motor neurons. It is important that the vectors will be live (but replication-incompetent) viruses, which have their own mechanisms of intracellular transport. We have engineered and expressed the fusion proteins, and have demonstrated that both ends—*i.e.*, the binding domain at the C-terminal end, and the SAv moiety at the N-terminal end—are functionally intact. They can bind to the ganglioside GT1b (a binding site for authentic botulinum type A holotoxin), motor neurons, several neuronal cell lines and neuromuscular junctions, and also bind directly to biotin and to biotinylated reporter molecules. 

### 1.1. ALS and SMA-Pathogenesis and Potential Therapeutic Agents

Recent studies have identified a variety of potentially pathogenic mechanisms and substances that are thought to contribute to motor neuron damage in ALS [2,5,6], and in spinal muscular atrophy (SMA) [7]. In ALS, these factors include glutamate excitotoxicity and abnormalities of glutamate transporters [8,9], mitochondrial abnormalities [10,11], accumulation of neurofilaments [12], protein aggregation [13], misfolding of proteins [6], proinflammatory cytokines [14], microglial activation [15], inflammation, oxidative damage [16,17], or increased intracellular calcium [18]. Five to 10% of ALS patients have a dominantly inherited form of the disease, which is phenotypically indistinguishable from sporadic ALS [19,20,21]. Approximately 20% of familial cases (1 to 2% of all ALS) are caused by mutations of superoxide dismutase 1 (SOD1), and well over 100 different ALS-causing mutations of SOD 1 have been identified [5]. Transgenic mice with human SOD mutations such as G-93-A have been intensively studied, and the mutant SOD is thought to produce toxic effects that represent a "gain of function", rather than a lack of SOD function [13]. Transgenic SOD 1 mice have been used in numerous pre-clinical therapeutic trials, some of which have been encouraging (see below). Most recently, mutations of the RNA-metabolizing protein, TDP-43, have been reported not only in familial cases of ALS [22], but also in sporadic cases [23,24]. Although the relative roles of these possible pathogenic factors undoubtedly differ in individual patients, it is likely that a cascade of events that may have common characteristics in different individuals results in the loss of motor neurons, chiefly by an ultimate process of apoptosis [25,26,27]. The goal of treatment of ALS is to prevent the death of motor neurons by interrupting this cascade at one or more points. The concept that the pathogenesis of ALS involves both non-neuronal and neuronal cells in the CNS, and is likely not to be strictly “cell autonomous” has been documented by a variety of experiments in mutant SOD transgenic mice [5,28]. 

In contrast to sporadic ALS, the underlying genetic abnormality in SMA is now known to involve loss of the *SMN 1* (survival motor neuron 1) gene. The clinical severity of SMA is influenced by the copy number of a similar, but less critically effective gene—*SMN 2* [7]. 

Knowledge of these pathogenic mechanisms has provided clues suggesting several attractive candidate treatment strategies [29,30]. Many of the candidates have been tested in SOD1 mutant mouse models of familial ALS (FALS), with resultant slowing of the course of the disease (for an excellent review, see Turner [31]). Some of the agents that have been shown to slow the onset or prolong the course of ALS in SOD mutant mouse models, include cyclooxygenase 2 inhibitors [32], insulin-like growth factor (IGF1) [33], certain neurotrophic factors such as BDNF (brain derived neurotrophic factor) and GDNF (glial derived neurotrophic factor) [34], and VEGF (vascular endothelial growth factor) [35]. The anti-apoptotic agent ZVAD/fmk delivered *via* the cerebral ventricles [14], or overexpression of the anti-apoptotic gene BCl-2 [36] have slowed the course of FALS in mice. The baculoviral anti-apoptotic p35 protein has prevented neurotoxic agent-induced cell death of cultured human neurons [37]. However the only treatment that has provided statistically definite, albeit minimal, benefit in human ALS is riluzole, which reduces neuronal glutamate release. 

Encouragingly, SMA has responded favorably both in animal models and in preliminary trials in humans to enhanced expression of the alternative *SMN* gene *SMN 2* induced by treatment with histone deacetylase inhibitors [7,38]. Delivery of the *SMN 1* gene to mice with a model SMA disease can rescue them by intravenous injection of adeno-associated virus 9 (AAV9) carrying the *SMN1* gene, but only if it is administered on postnatal day 1. If it is given on postnatal day 5 there is only partial benefit, and no benefit is realized if given on day 10 [39] This suggests that there is a very limited window in which IV injection of AAV9 can target neurons in sufficient numbers for benefit in SMA. Presumably true replacement of the *SMN1* gene could have a profound beneficial effect in this disorder. It is likely that one or more of these treatment modalities, *if provided* *efficiently to the motor neurons*, could have more profound therapeutic effects. There is widespread belief, based on a wealth of experimental evidence [31,40], that gene therapy, if adequately delivered by viral vectors, will provide the best opportunity for treatment of these motor neuron diseases.

### 1.2. Accessing Motor Neurons—The Problem

One of the most frustrating problems in devising treatments for ALS or SMA is the difficulty of delivering therapeutic agents or related genes of interest to the motor neurons. Systemic delivery of most agents is unlikely to achieve sufficient access to motor neurons, and this limitation may be the cause of failure of many of the therapeutic trials in human ALS, whereas relatively higher doses may have shown some benefit in mice. Direct injection of substances or cells into the spinal cord [41], infusions into the spinal fluid [14], and extensive injections of viral vectors throughout skeletal muscles [33] are possible but difficult methods of delivering agents or transferring genes to motor neurons. However, it has been shown that certain viral vectors (Adenovirus, AAV, and lentivirus) do undergo retrograde transport, provided that they enter the motor nerve terminals [33,42]. For the most part, this is relatively inefficient, because only a small proportion of the viral vectors injected into the large bulk of the skeletal muscle can reach the very small region of the motor nerve terminals. Pseudotyping a lentiviral vector with an equine rabies (EIAV) capsid enhanced entry into neurons (but not specifically motor neurons) [35]. The fact that these viruses are known to be carried to the cell bodies of motor neurons by retrograde transport [33,35,39,42] is a key element of the present approach, which is designed to direct their entry into the motor nerve terminals. 

### 1.3. Viral Vectors

All three types of viral vectors that have been used experimentally (AdV, AAV, and lentivirus) have advantages and disadvantages. AdV is the most readily produced, but is known to induce immune responses in host animals (though “gutted” AdV deleted of most viral DNA sequences are reportedly less immunogenic [43]). AAV is somewhat more difficult than AdV to produce in high quantities, but is virtually non-immunogenic, is known to produce long-lasting gene expression, and is the only viral vector that is currently potentially approvable for human use. Lentiviral vectors also have the advantage of long-term expression, but the fact that their integration in the genome may occur at unwanted sites poses a risk of the development of cancer, and the re-appearance of replication competent lentiviruses [44,45,46]. It is therefore less likely to be approved for human use in the forseeable future. 

### 1.4. Agents that Enter Motor Neurons Specifically

Only a few substances specifically bind to and enter motor neuron terminals, and are capable of providing access to the cells. These include poliovirus, tetanus toxin, rabies virus—with lesser specificity, and botulinum toxin. Since most individuals in the developed world have been immunized against poliovirus and tetanus toxin, these are not useful. Our strategy in the research described here is to make use of the binding and translocation domain of type A botulinum toxin heavy chain as a “Trojan horse” to facilitate delivery of viral vectors carrying potentially therapeutic genes into motor nerve terminals, where they enter the endosomal compartment, and are subjected to its acid milieu, and are processed. The viral vector is then liberated, and presumably will be carried by retrograde transport to cell bodies of motor neurons. It should be emphasized that live viral vectors do not behave like inert protein cargoes, but can coopt cellular mechanisms for intracellular transport.

### 1.5. Botulinum Toxin

**Figure 1:**
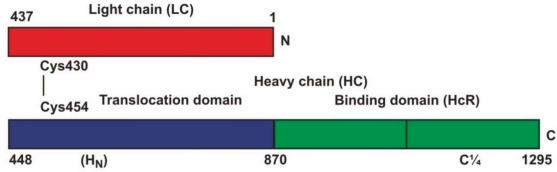
Botulinum Neurotoxin A: The full length binding domain (HcR) extends from residues 870–1,295, and is labeled green. The “C quarter” starts from the vertical line in the binding domain, and occupies the last portion (C’ end) of the heavy chain.

Botulinum Type A holotoxin (BoNT/A) consists of a heavy chain (100 kDa) and a light chain (50 kDa), joined by a disulfide bond [47] (Figure 1). The heavy chain’s C-terminal domain binds specifically to cholinergic motor terminals, *via* gangliosides (predominantly GT1b and also GD1a), but also requires binding to a protein co-receptor, SV2 [48,49]. After binding to the motor nerve terminal, the heavy chain (or its binding domain) undergoes endocytosis [50], and enters the endosomal compartment, which is highly acidified [47]. In the case of the native holotoxin, the toxic light chain is cleaved off, and enters the cytoplasm [51], where it achieves its toxic effect by cleavage of SNAP-25, a protein essential for release of the neurotransmitter acetylcholine from the nerve terminal. Botulinum toxin is the most poisonous poison known, with LD_50_ values in humans estimated to be in the 0.1 to 1 ng/Kg range [52] This extraordinary sensitivity is due to the exquisite specificity of binding to the motor nerve terminals, as well as the efficiency of blockade of ACh release from nerve terminals by the toxic moiety, with resultant paralysis. The proposed research will make use of the remarkable specificity of binding of the heavy chain's C-terminal binding domain, to re-direct viral vectors (which can be designed to carry potentially therapeutic genes), to enter the motor nerve terminals efficiently. 

The entry of the translocated viral vectors into the endosomal compartment which is highly acidic reproduces the natural trafficking pathway of AAV and AdV [53,54], and is expected to result in appropriate processing of the redirected viral vectors. The processed viral vectors are then released, to enter the retrograde transport pathway, and reach the motor neuron cell bodies.

In general, neither the holotoxin nor the binding domain of botulinum toxin itself undergoes retrograde transport. Therefore we do not anticipate that the binding domain of botulinum will participate in retrograde transport of the viral vector (see below). However, the role of the binding domain in this strategy is to bind to motor nerve terminals, translocate across the terminal membrane together with the biotinylated viral vector that is linked to SAv of the fusion protein, and enter the endosomal vesicle. Acidification within the endosomal compartment will then release the viral vector which can thereby enter the retrograde transport pathway, utilizing its natural mechanisms.

### 1.6. Retrograde Transport of AdV and AAV

Both AdV and AAV have been shown to undergo retrograde transport after intramuscular injection [33], and Miagkov & Drachman, unpublished studies. The intracellular trafficking of native AdV and AAV begins with binding to the surface receptor. They are rapidly endocytosed, and move to early endosomes within minutes [53,55,56]. Acidification of the endosomes is required for release from the endosomal compartments. AdV appears to be released rapidly, whereas AAV may enter the late endosomal compartment before release [53,55]. Details of the intracellular trafficking are not completely clear, but the viruses reach the nucleus or perinuclear region of infected cells. Genes delivered by AAV either remain episomal, or may be integrated in chromosome 19, while genes delivered by AdV are not integrated into the genome. The efficiency of retrograde transport of viral vectors that have been injected into skeletal muscles is generally quite low, presumably because only a very small proportion of the viral load presented to the large bulk of the skeletal muscle enters the small domain of the motor nerve terminals. Therefore, treatment of ALS patients by intramuscular virus injections would require both large amounts of viral vectors and numerous injections into skeletal muscles. Based on the proposed research, the specificity of entry of the BoNT-SAv redirected viral vectors into motor nerve terminals should greatly increase the efficiency of transduction of motor neurons. Prior studies on viral vectors that have been re-directed to cellular receptors show that the efficiency is increased by 50–100-fold [57,58].

## 2. Methods and Results

*The Fusion Proteins* In order to construct the fusion proteins, we required authentic genes both for SAv and for the binding domain of Botulinum Toxin Type A. 

### 2.1. Streptavidin

The gene for SAv was a gift of Dr. Jody Schultz [59], and was provided in the plasmid PEX 318. We obtained the full length SAv gene by PCR, using appropriate primers with NdeI restriction sites at both ends, and cloned the gene into a pET15b expression plasmid (Novagen), which fuses a 6-histidine tag to the N terminus for purification. We expressed the SAv in E. Coli BL-21, and purified it on a nickel column (Ni-NTA His-Bind® Resins Novagen Co) by elution with imidazole. The purified SAv bound quantitatively to biotin-coated ELISA plates (Thermo-Scientific, Rockford, IL, USA), as compared with commercial grade SAv.

### 2.2. Binding Domain of Botulinum Type A

**Figure 2:**
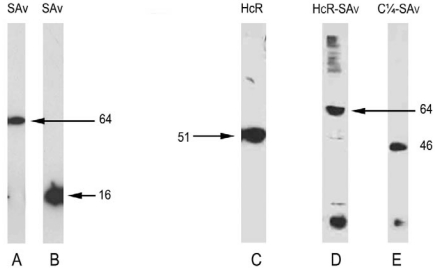
Western blots showing proteins used in these experiments. A: Streptavidin, non-denatured gel showing the tetrameric form of expressed SAv at ~64 kD; B: reduced (monomeric) form of SAv at ~16 kD; C: HcR expressed in pET28a at ~51 kD; D: Partially denatured gel: HcR-SAv fusion protein expressed in pET15b at 64 kD is denatured monomeric HcR-SAv. The lowest band is separated denatured SAv. The higher M. W. bands are multimeric HcR-SAv that were not denatured. The natural state of HcR-SAv in solution is tetrameric; E: Denatured gel: C¼-SAv fusion protein expressed in pET15b at 46 kD.

We obtained the gene for Botulinum Toxin Type A heavy chain binding domain (HcR) that was codon-optimized, in an expression plasmid (pET28a) as a generous gift from Dr. J. Barbieri (Medical College of Wisconsin) [60] (Figure 1). Because of the unusual codons in BoNT, we used BL-21Codon Plus RIPL^®^ competent E. Coli (Agilent-Stratagene) as an expression vector, with greatly improved yields as compared with the standard BL21 E Coli vector. The Codon Plus E. Coli transformed with this plasmid was grown up at 37 °C to a cell density 0.6–0.9 at A 600nm, and expressed by overnight shaker incubation with IPTG 100 µM, at the unusually low temperature of 16 °C. The cells were harvested, and broken by passage 2 or 3 times through a French press. The lysate, which contained the great majority of the expressed protein with a poly-His tag, was purified as above on a Ni column by elution with imidazole. This “full length” binding domain preparation (HcR) consisted of approximately the C-terminal half of the heavy chain, and appeared as a single band on Western blot at approximately 51 kD (Figure 2). We obtained an average of more than 10 mg of purified protein from 1 L of cells. As described below, the protein behaved appropriately for the botulinum binding domain: It bound to the ganglioside target for botulinum toxin (GT1b) in an ELISA assay, to N2A (mouse neuroblastoma) cells, to chick embryo primary motor neurons in cultures, and to NG108-15 (mouse neuroblastoma x rat glioma) cells, but did not bind to NG-CR-72 cells, (a parallel cell line that lacks complex ganglioside expression). Moreover, it blocked the ability of authentic botulinum Type A holotoxin to cleave SNAP-25 in chick embryo motor neurons. Finally, the HcR binding domain bound *in vivo* to mouse diaphragm neuromuscular junctions.

### 2.3. Construction of Fusion Proteins with “Full Length” HcR and “C-quarter” Binding Domain

We excised the HcR from the pET28a plasmid with the restriction enzymes XHO1 and BamH1, and amplified it by PCR. We also amplified the C-terminal half of the HcR, using a different upstream primer, to obtain a DNA sequence (referred to as the C¼) that was 654 base pairs shorter than the "full length" HcR, but contained the relevant C-terminal part of the binding domain. Each of these constructs was then ligated into the pET15b plasmid, as a preliminary step in the construction of the fusion proteins. We then transformed the BL-21Codon Plus RIPL^®^ E. Coli with these plasmids, and expressed them as described above for the original HcR protein. The HcR and C¼ proteins expressed similarly to the original HcR, and behaved similarly in all respects, as expected for the binding domains of BoNT Type A.

To prepare the fusions of the Botulinum binding domain proteins with SAv, we excised the "HcR" and “C¼” sequences with the same restriction enzymes, amplified them by PCR, and ligated them into the previously prepared SAv expression pET15b plasmid, 3’ to the SAv gene (with an intervening VSV [vesicular stomatitis virus] signal sequence as a tag). 

Expression of the fusion proteins was carried out as for the original HcR protein. The average yield of purified full length HcR-SAv or of C¼-SAv fusion proteins was 4 to 8 mg per L of cells. The two fusion proteins behaved similarly in binding assays, as described below. However, the “full length” HcR-SAv proved to be a little more potent in blocking cleavage of SNAP-25 by authentic botulinum Type A holotoxin. Both the fusion proteins bound to biotin in the ELISA assay.

### 2.4. Functional Activity of the Fusion Proteins: Testing and Results

The fusion proteins—both the HcR-SAv and the C¼-SAv—were tested *in vitro* for their ability to bind to neuronal cells, as follows:

### 2.5. Chick Embryo Primary Motor Neurons

The lumbar regions of chick embryos at 5½ to 6 days of incubation age were dissected free, and the cells were isolated and cultured as described [61]. After culture for 3 to 6 days, the cells were differentiated, and were used for testing the ability of expressed proteins (HcR, HcR-SAv, C¼-SAv) to bind to the cells, and to protect against cleavage of SNAP-25 by authentic BoNT Type A. For binding studies they were grown on cover slips, and for cleavage studies they were grown in 24 well culture plates.

### 2.6. Binding to Motor Neurons

**Figure 3:**
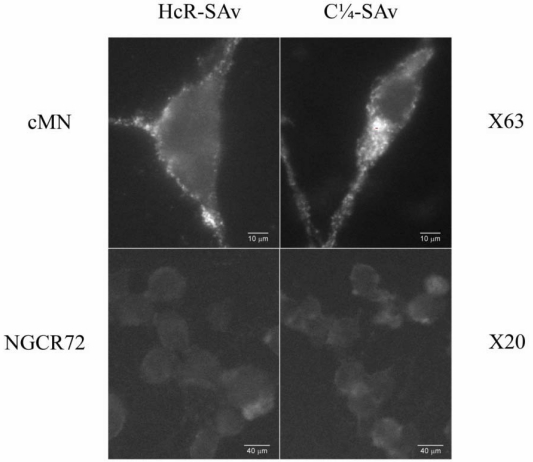
Binding of Fusion Proteins to Chick Motor Neurons. The fusion proteins HcR-SAv and C¼-SAv bound to chick motor neurons in culture, as shown in the upper two panels. They did not bind to the NG CR72 cells, which lack complex gangliosides (lower panels).

Each of the expressed proteins was added at a final concentration of 100 to 800 nM in culture medium and incubated at 4 °C for 60 min. Binding was detected by fluorescence microscopy, using mouse monoclonal antibodies directed to the specific tags (Figure 3). For HcR we used mouse monoclonal anti-Flag tag, followed by fluoresceinated goat-anti-mouse antibody. For the fusion proteins we used rabbit anti-VSV antibody, and fluoresceinated goat anti-rabbit antibody.

### 2.7. Blocking SNAP-25 Cleavage [62]

**Figure 4:**
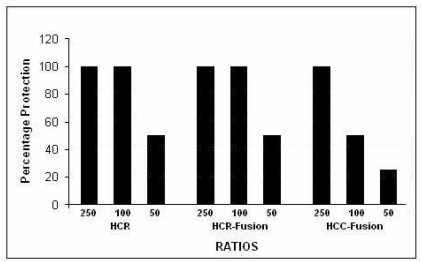
Cleavage of SNAP-25 by authentic Botulinum type A holotoxin complex was blocked by HcR, HcR-SAv fusion protein and C¼-SAv fusion protein (% protection).

Each of the expressed proteins was added to wells containing chick motor neuron cultures together with 1.5 µg Botulinum Type A holotoxin complex (generously provided by Dr. Eric Johnson), at a concentration of 0 to 250-fold that of the toxin, and incubation was continued for 3 h at 37 °C. Western blots of the cultures were stained with antibody to SNAP-25 (Covance clone SMI 81, diluted 1:1000). All three of the expressed proteins blocked SNAP-25 cleavage, consistent with their ability to bind to botulinum-specific binding-sites (Figure 4). The C¼-SAv produced less complete blockade at the lower multiples, possibly because its smaller size did not provide as broad spatial coverage of the botlinum binding sites. Depending on the number of culture wells available, single or duplicate wells were used for each concentration of expressed protein in different experiments. These blocking experiments were repeated 8 times with various batches of expressed proteins, with closely similar results. 

### 2.8. Binding to and Uptake by Neuronal Cell Lines

The N2A (mouse neuroblastoma) cell line was obtained from American Type Culture Collection, and differentiated by the addition of retinoic acid. NG108-15 cells (mouse neuroblastoma x rat glioma) cells and NG-CR72 cells (parallel cell line lacking complex gangliosides) were generously provided by Dr. R. Schnaar, and cultured as described [63]. Each of the expressed proteins was added in culture medium at a final concentration of 100 to 800 nM, and incubated at 4 °C for 60 min. Binding of the proteins was detected by fluorescence microscopy, as above. Each of the three proteins bound to the N2A and NG108-15 cells, but not to NG-CR72 cells.

**Figure 5:**
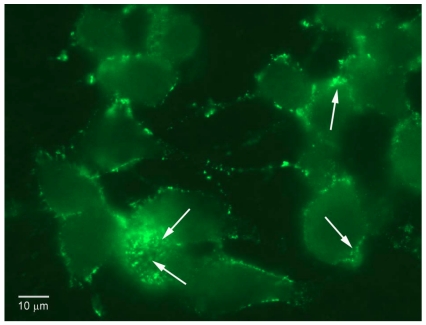
Endocytosis of HcR-SAv by N2A cells. The cells were incubated with HcR-SAv at 37 °C for 1 h, fixed and permeabilized, and stained for the VSV tag. Note internalization of the HcR-SAv (arrows).

To evaluate endocytosis of the proteins by the neuronal cells, we used the larger N2A cells, in which cytoplasm was more readily visualized than in the other cell lines. The N2A cells were incubated with each of the proteins at 37 °C for 1 h. The cells were then permeabilized with 0.1% Triton X-100, and stained as above. The results showed entry of the proteins into the N2A cells (Figure 5).

### 2.9. Binding to Ganglioside GT1b

Since BoNT Type A is known to bind to the ganglioside GT1b, we evaluated the ability of the expressed proteins to bind to this ganglioside in an ELISA assay. ELISA plates were coated with GT1b (Sigma, 1:100 of a 1 mg/ml solution in methanol, overnight, blocked with BSA), and serial dilutions of each of the expressed proteins were added to duplicate wells. Binding was evaluated by the addition of Goat anti-His-HRP (Roche) developed with TMB substrate (BioRad). The results demonstrated strong and essentially equal binding of all three proteins to GT1b.

### 2.10. Binding to Biotin

In order to evaluate the ability of the fusion proteins to bind biotin, we carried out ELISA assays, similar to those for pure SAv, using biotin-coated ELISA plates (Thermo-Scientific, Rockford, IL, USA). In these assays, the amount of binding was compared with binding of purified SAv. Both HcR-SAV and C¼-SAv bound to the biotin. We calculated the efficiency of binding based on the relative amount of Streptavidin in each of the fusion complexes. The HcR-SAv fusion protein bound at the level of 67%, while the C¼-SAv bound at 65.5%.

### 2.11. Binding to Neuromuscular Junctions in vivo

**Figure 6:**
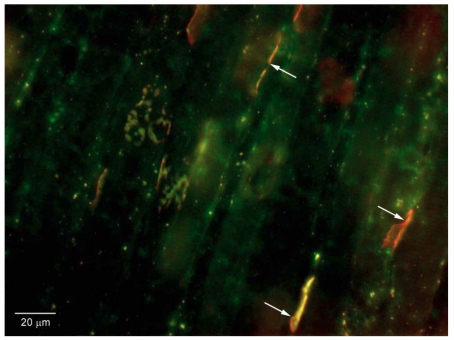
Binding of fusion protein HcR-SAv with Biocytin tag to mouse diaphragm neuromuscular junctions. Biocytin is green-yellow; post-synaptic ACh receptors are stained with Texas red α-BuTx. Note co-localization of red and green at N-M junctions (three dually-stained N-M junctions are marked with arrows).

To evaluate the ability of the proteins to bind to motor nerve terminals, we injected each purified protein directly into both pleural cavities of C57Bl/6 mice (protocol approved by the Johns Hopkins Animal Care and Use Committee), using a method similar to that previously described [64]. The mice were anesthetized with isofluorane, and 10 to 15 µg of each of the proteins in 75 µL of PBS was injected in each pleural cavity. The anesthetized mice were maintained in a vertical position for 1 h to allow the fluid to gravitate to the diaphragm, and they were killed by cervical dislocation. The diaphragms were removed, washed repeatedly with PBS, pinned at resting length on a silastic platform under a dissecting microscope, and then fixed with 2% paraformaldehyde. After thorough washing with PBS, segments containing the motor endplate zones were cut radially, placed on coverslips, immunostained as above, counterstained with Texas red conjugated α-bungarotoxin to mark the post-synaptic regions, and examined by fluorescence microscopy and/or confocal microscopy. The results showed that each of the three proteins: HcR, HcR-SAv, and C¼-SAv bound to the neuromuscular junction and essentially co-localized with the Texas red (Figure 6). This indicated that the ability of both the HcR and its SAv—fused products to bind *in vivo* was intact. 

### 2.12. Ability of Fusion Proteins to Bind while Carrying Biotinylated Fluorophore

We then evaluated the ability of the fusion proteins to bind while carrying a biotinylated protein at the SAv terminal end. For this purpose, we obtained “Biocytin”®, a conjugate of biotin and the fluor Alexa 488 (Molecular Probes). Biocytin Alexa 488 was incubated for 1 hr at room temperature at a 1:1 molar ratio with each of the fusion proteins, and unbound Biocytin was removed by spin filtration (Zeba Spin Desalting Column, Thermo Scientific). The results showed excellent binding of HcR-SAv-Biocytin and C¼-SAv-Biocytin both to neuronal cells in culture and to neuromuscular junctions *in vivo*. This indicated the ability of the fusion proteins to bind while carrying biotinylated loads.

## 3. Discussion and Conclusions

As described above, we have succeeded in engineering and expressing a heterobifunctional protein, designed to carry biotinylated viral vector cargoes specifically into motor nerves. As indicated, a fusion protein consisting of the binding domain or a C-terminal fragment of it, derived from the heavy chain of Botulinum Toxin Type A, fused to streptavidin, can be expressed in substantial quantities, and is functionally capable of binding to and entering neurons and motor nerve terminals, and binding to biotin and biotinylated molecules. Moreover, the fusion proteins function equally well when carrying biotinylated “cargoes” consisting of protein tags. The goal is to utilize this reagent to transport biotinylated viral vectors carrying genes of interest into motor neurons for the treatment of motor neuron diseases, including ALS and SMA. 

We anticipate that the biotinylated viruses—particularly AAV—will be transported into the lysosomal compartments of motor nerves, which are the sites that both botulinum toxin and the viral vectors normally enter. It should be emphasized that the vectors will be live (but replication-incompetent) viruses, which have their own mechanisms of intracellular transport. However, this report is a “work in progress”, and there is more work to do in order to establish the efficacy and potential clinically therapeutic value of this strategy, using viral vectors.

Our next immediate goal is to demonstrate that the fusion proteins are capable of carrying biotinylated viral vectors into neuronal cells. Biotinylation of viruses can be achieved either chemically or by metabolic methods. There are numerous commercially available biotinylation reagents and kits that allow the conjugation of biotin to a variety of surface molecules, with intervening spacers of different lengths. Several of the biotinylation kits permit assessment of the degree of biotinylation. Alternatively, methods for metabolic biotinylation of AAV have been developed [65], and are efficient, gentle and regulatable. The Biotin Acceptor Protein (BAP) is inserted in the capsid of the AAV, and the AAV is then produced in HEK293 cells that are transduced with the biotinylating enzyme BirA, resulting in AAV with a capsid containing approximately 20 biotin molecules. Which of the biotinylation methods proves more effective for this purpose remains to be determined. Biotinylated AAV expressing a fluorescent tag (EGFP) will be used for the next steps. These biotinylated AAV vectors will be incubated with each of the fusion proteins at various ratios of virus: fusion protein. The resultant fusion protein-virus complexes will be used to evaluate specificity of gene expression in neuronal cells, initially *in vitro*.

For this purpose, we will utilize the same neuronal cell lines that we have used in the experiments described above, *i.e.*, N2A cells or NG108-15 cells. The cells will be incubated with limiting amounts of the fusion protein-virus complexes, or with an equivalent amount of non-complexed AAV. The read-out will be expression of EGFP. We anticipate that AAV complexed with the targeting fusion proteins will result in much more efficient gene expression as compared with the native AAV.

The results of the above experiments will serve as a guide for the next *in vivo* phase. This will be designed to evaluate the efficiency of the fusion-complexed AAV in carrying genes of interest by retrograde transport to the motor neuron cell bodies. As in our *in vivo* binding experiments described above, we will inject fusion-complexed AAV with EGFP genes into the pleural cavities of mice. After appropriate intervals, the mice will be killed, and the cervical spinal cord segments supplying the phrenic nerves will be examined for expression of EGFP. As above, we will compare the efficiency of expression of EGFP by the fusion-complexed AAV with that of native AAV. We will evaluate the intervals required for release of the AAV from the lysosomal compartments, and for retrograde transport. We also expect to test the expression of AAVs that are delivered in the form of complexes *via* the intravenous route. If systemic administration is successful, it would greatly enhance the ability to provide therapeutic genes to virtually all the motor neurons, rather than only those that can be reached by local injection into the pleural cavities, or muscles.

Of course, the ultimate goal of this work is to develop and test candidate genes for the treatment of ALS and SMA. In this phase, we will produce AAV vectors loaded with the gene(s) of interest, and deliver them in the form of fusion protein-AAV complexes. Depending on the optimum route of delivery, we will inject fusion protein-AAV complexes intrapleurally, intramuscularly, or intravenously, using test models of ALS such as the G93A SOD1 transgenic mouse. The effects will be evaluated as we and others have done in the past [32], in terms of the survival, the strength, and the histopathology of the mice. We will also test the effects of delivery of the gene SMN at various postnatal stages to SMA mice lacking the SMN gene [39].

There are many advantages to this strategy. The production of the fusion proteins is now fairly routine, and the yields are abundant. The AAV vectors are known to be carried to motor neuron cell bodies by retrograde transport, provided they enter the motor nerve terminals. AAV vectors are the only viral vectors likely to be approved for human use in the foreseeable future. Although they require expertise in production, the effort would be highly worthwhile. If successful, this strategy will allow the delivery of a variety of different potentially therapeutic genes to motor neurons fairly simply, by varying the gene inserted into the AAV. We do not expect that a single gene will prove curative, except perhaps in the case of SMA. An advantage of this strategy is its adaptability to allow testing of many different candidate therapeutic genes, with only relatively minor alterations in the protocols. Further, if the targeting complexes succeed in delivering genes that are introduced intravenously to motor neurons, that would allow the widespread distribution of therapeutic genes to motor neurons throughout the neuraxis. 

Admittedly, there is much to do before this strategy will be ready for treatment of human diseases. However, we are well on the path, and the direction for future development of this method is clear. Undoubtedly, there will be unforeseen problems to solve along the way. However, this strategy takes advantage of the exquisite specificity and sensitivity of botulinum toxin for therapy of what are now untreatable diseases.
